# Nanoelectromechanical Infrared Spectroscopy with In Situ Separation by Thermal Desorption: NEMS-IR-TD

**DOI:** 10.1021/acssensors.2c02435

**Published:** 2023-04-17

**Authors:** Niklas Luhmann, Robert G. West, Josiane P. Lafleur, Silvan Schmid

**Affiliations:** † Institute of Sensor and Actuator Systems, TU Wien, Gusshausstrasse 27-29, 1040 Vienna, Austria; ‡ Invisible-Light Labs GmbH, Taubstummengasse 11, 1040 Vienna, Austria

**Keywords:** NEMS, IR spectroscopy, temperature-programmed desorption, sample separation, thermogravimetric analysis, isothermal desorption

## Abstract

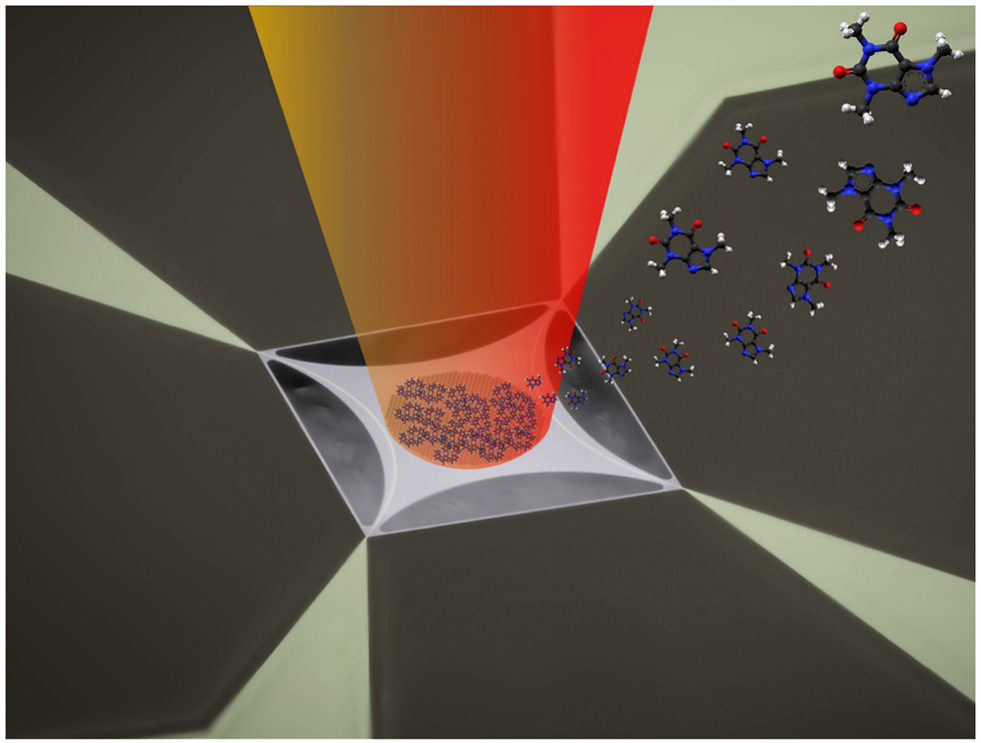

We present a novel method for the quantitative analysis of mixtures of semivolatile chemical compounds. For the first time, thermal desorption is integrated directly with nanoelectromechanical infrared spectroscopy (NEMS-IR-TD). In this new technique, an analyte mixture is deposited via nebulization on the surface of a NEMS sensor and subsequently desorbed using heating under vacuum. The desorption process is monitored *in situ* via infrared spectroscopy and thermogravimetric analysis. The resulting spectro-temporal maps allow for selective identification and analysis of the mixture. In addition, the corresponding thermogravimetric data allow for analysis of the desorption dynamics of the mixture components. As a demonstration, caffeine and theobromine were selectively identified and quantified from a mixture with a detection limit of less than 6 pg (about 30 fmol). With its exceptional sensitivity, NEMS-IR-TD allows for the analysis of low abundance and complex analytes with potential applications ranging from environmental sensing to life sciences.

Thanks to its ability to provide chemical fingerprints, infrared (IR) spectroscopy has found a wide range of applications. However, similar to other detection methods, the analysis of complex samples remains a challenge with IR spectroscopy. The postmeasurement deconvolution of complex spectral data with the help of dedicated mathematical algorithms, or more recently with artificial intelligence,^1,2^ is often necessary. A solution to the analysis of complex samples consists of hyphenating the detection technique with a separation step upstream, such as liquid or gas chromatography (LC/GC). This method is widely used for mass spectrometry (MS), where LC and GC are used extensively to separate analytes prior to detection. GC and LC have also been successfully combined with Fourier transform IR spectroscopy (FTIR).^3,4^ For online analysis, GC or LC can be interfaced with FTIR by means of IR-transparent flow cells.^5−8^ However, compared with MS, online GC/LC-FTIR suffers from significantly reduced sensitivity. Therefore, offline methods such as cryogenic trapping (matrix isolation) GC-FTIR,^9^ direct-deposition GC-FTIR,^10,11^ and solvent elimination LC-FTIR^4^ have been developed. They continuously collect the analyte fractions for postmeasurement analysis, which allows for an extended data acquisition time reaching subnanogram sensitivities when used in conjunction with highly sensitive cryogenically cooled IR detectors.^12,13^

Over the past few years, nanoelectromechanical infrared spectroscopy (NEMS-IR) has demonstrated that low picogram sensitivities can be achieved without cryogenic cooling for the analysis of various samples such as polymer nanoparticles,^14^ micelles,^15^ explosives,^16^ pharmaceutical compounds,^17,18^ and polymer thin films.^19^ In this approach, an analyte is collected or deposited on a nanoelectromechanical resonator and subsequently irradiated with IR light. The resulting wavelength-specific photothermal heating of the analyte causes frequency detuning of the nanoelectromechanical resonator that is proportional to the absorbed IR power. However, just like with traditional IR techniques, the analysis of complex samples with NEMS-IR remains a challenge, and the technique has, to date, been demonstrated only as a proof of concept for single analytes. The hyphenation with GC/LC would technically be possible by continuously collecting analyte fractions on an array of individual NEMS detectors. This approach would, however, suffer from the same complexity that has hindered the proliferation of offline GC/LC-FTIR methods.^20^

Here, we introduce NEMS-IR-TD featuring an improved resonator design and *in situ* sample separation by thermal desorption, similar to temperature-programmed desorption (TPD). TPD is typically used as an analysis tool to study adsorbate–surface interactions,^21^ decomposition,^22,23^ and catalytic processes.^24^ However, it can also be used as a separation method.^25^ Here, the analyte is collected on the surface of the nanomechanical resonator by an aerosol-based sampling method. As the temperature is increased, the sample components desorb in order of decreasing volatility. In NEMS-IR-TD, the desorption process is monitored twofold, first by continuously recording IR spectra every 2 min and second by measuring the mass loss in real time. Both the spectral and mass data can be obtained from the frequency detuning of the NEMS resonator. The resulting time-resolved IR spectra can readily be decomposed by singular value decomposition (SVD) and a global analysis adapted from the field of ultrafast spectroscopy.^26−29^ Additionally, the mass data allow for the simultaneous study of the components’ desorption dynamics as has been demonstrated with NEMS paddle resonators^30^ or surface acoustic wave detectors.^31^ As a demonstration of NEMS-IR-TD, binary mixtures of two chemically similar compounds, caffeine and theobromine, were analyzed with limits of detection of 5.7 and 4.9 pg, respectively (∼30 fmol). In contrast to hyphenation, *in situ* separation eliminates the necessity to transfer the sample from the separation device to the detection device, minimizing sample losses, dilution, and contamination. With its exceptional sensitivity and variety, this method allows for the analysis of low-abundance and complex analytes with potential applications ranging from environmental sensing to life sciences.

## Experimental Section

### Experimental Setup

**Figure 1:**
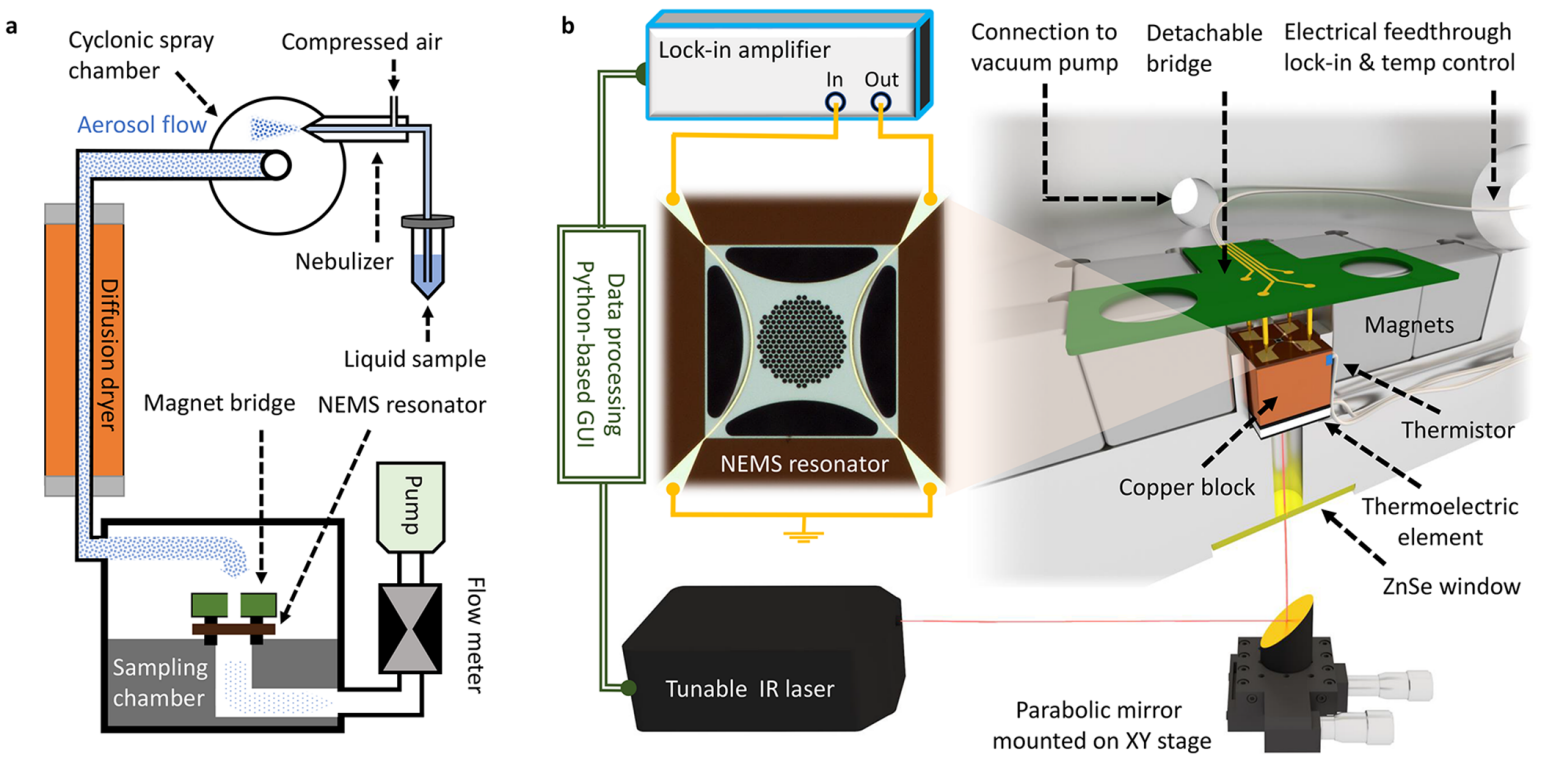
Schematic drawing of the experimental setup consisting of (a) a sampling and (b) an analysis module. At the core of the setup a low-stressed and perforated resonator made of 50 nm silicon nitride is used. The resonator is designed to provide a high thermal response and enable aerosol sampling, and it comprises two electrodes for an integrated electrical transduction. For sampling, the liquid analyte is nebulized, dried, and flushed through the perforations of the resonator. In the analysis module, the resonance can be continuously tracked and driven by a phase-locked loop by exploiting the Lorentz force and induced voltage of two independent gold electrodes placed along the resonators’ rim. A spring-loaded contact bridge provides the electrical connection and fixation of the chip on top of a copper block, which is thermally connected to a thermoelectric element for precise control of the resonator temperature. The spring-loaded contact allows for a fast loading and unloading of NEMS chips. The chamber is connected to a turbo pump to provide an operational vacuum of as low as 10^–5^ mbar. A dedicated Python-based user interface allows for simultaneous control of the tunable IR laser and monitoring of the resonance frequency for automated spectral acquisition.

Figure 1 shows a schematic of the NEMS-IR-TD setup. The setup is inspired by previous studies.^17^ The system was modified to allow for easy NEMS sensor chip exchange without wire bonding and fitted with temperature control of the NEMS sensor chip in the range of 0 to 70 °C. First, the analytes are sampled from solution onto the NEMS resonator by means of an aerosol-based method,^17,32^ as depicted in Figure 1a. Second, the chip is transferred to the analysis chamber, depicted in Figure 1b, to perform dispersive photothermal IR spectroscopy with a tunable quantum cascade laser (QCL, MirCat Daylight Solutions) which covers the spectral region from 1779 to 1122 cm^–1^. Each IR spectrum is recorded in a single continuous wavelength sweep. The chip is directly held in place on top of a temperature-controlled and calibrated copper block (Supporting Information Figure S4) and electrically connected with spring-loaded probes, which allows for quick loading and unloading. The photothermal IR analysis is performed in vacuum at a pressure below 10^–4^ mbar to reduce air damping of the resonator, prohibit heat transfer through convection, and promote a steady desorption of the sample. A Python-based graphical interface was developed to control the QCL’s wavelength range, speed, and repetition interval while simultaneously monitoring the oscillation frequency of the NEMS resonator driven by a phase-locked loop. The graphical interface makes it possible to perform fully automated spectra acquisition over a defined time period.

### Nanomechanical Resonator Design

All resonators used in this work were fabricated using standard clean room techniques from 50 nm thin silicon nitride with a tensile stress of about 100 MPa, deposited on 4 in. Si wafers by low-pressure chemical vapor deposition (LPCVD) (Hahn-Schickard, Germany). The resonators used throughout the analyses featured a perforated trampoline suspended over a square orifice of 500 and 1000 μm side lengths.

### Sample Preparation and Sampling

All analytes in this work were sampled from a liquid solution using standard aerosol techniques by a setup developed in house (Figure 1(a)). The aerosol was generated by a pneumatic jet nebulizer (ESI, MicroFlow PFA-ST) with a self-aspiration capillary (20 μL min^–1^) and pressurized dry air at 3 bar. Smaller droplet selection was achieved by using a cyclonic spray chamber (Meinhard, ML148030). The remaining smaller droplets were dried and reduced to a particulate aerosol by passing it through a diffusion dryer (TOPAS DDU 570/L). In the final step, the dried aerosol was flushed in a separate chamber through the orifice and perforation of the resonator where it was sampled by inertial impaction and intersection.^17^ In the presented work, the crystalline xanthine compounds, caffeine (Sigma-Aldrich 42993-100MG) and theobromine (Sigma-Aldrich C0750-5G), were dissolved and diluted in Milli-Q water to a stock solution with a concentration of 50 to 62 μg mL^–1^. In order to minimize the surface adhesion of the compound to the resonator, the chips were additionally passivated with trimethylchlorsilane (Sigma-Aldrich, cat. no. 386529) following a protocol from Szkop et al.^33^

### Sample Mass Calculation

For small changes, the response to mass can be linearized by a first-order Taylor expansion, given by *R* = −1/(2*m_r*),^34^ where *m_r* corresponds to the resonator mass. However, in this work several nanograms of analyte were sampled, which is within the range of the resonator mass itself. Therefore, a third-order Taylor expansion was used to determine the equivalent mass load, given by
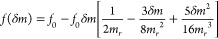
1where *f*(*δm*) is the resonance frequency of the loaded mass *δm* and *f*_0_ = *f*(*δm* = 0) of the unloaded resonator. In all presented analyses, the equivalent mass load of the measured compound was obtained from eq 1 by using the numerical solver from sciPy (Python) and *f*_0_ as the resonance frequency of the (unloaded) chip after each measurement.

### Spectra Acquisition and Data Postprocessing

All IR spectra in this work were acquired with the homemade graphical interface using a continuous sweep mode of the QCL set to a speed of 25 cm^–1^ s^–1^ (26 s per spectrum) and a repetition interval of 2 min to record spectro-temporal maps. The relative frequency change is obtained automatically by recording the resonance frequency *f*_0_ for 1 s prior to the laser emission and dividing −*δf* by *f*_0_ after the sweep is completed. In order to compensate for additional influences on the resonance frequency as the continuous rise due to desorption during a sweep and further influence by temperature, all spectra presented in this work were postprocessed using a homemade Python script.

## Results

**Figure 2:**
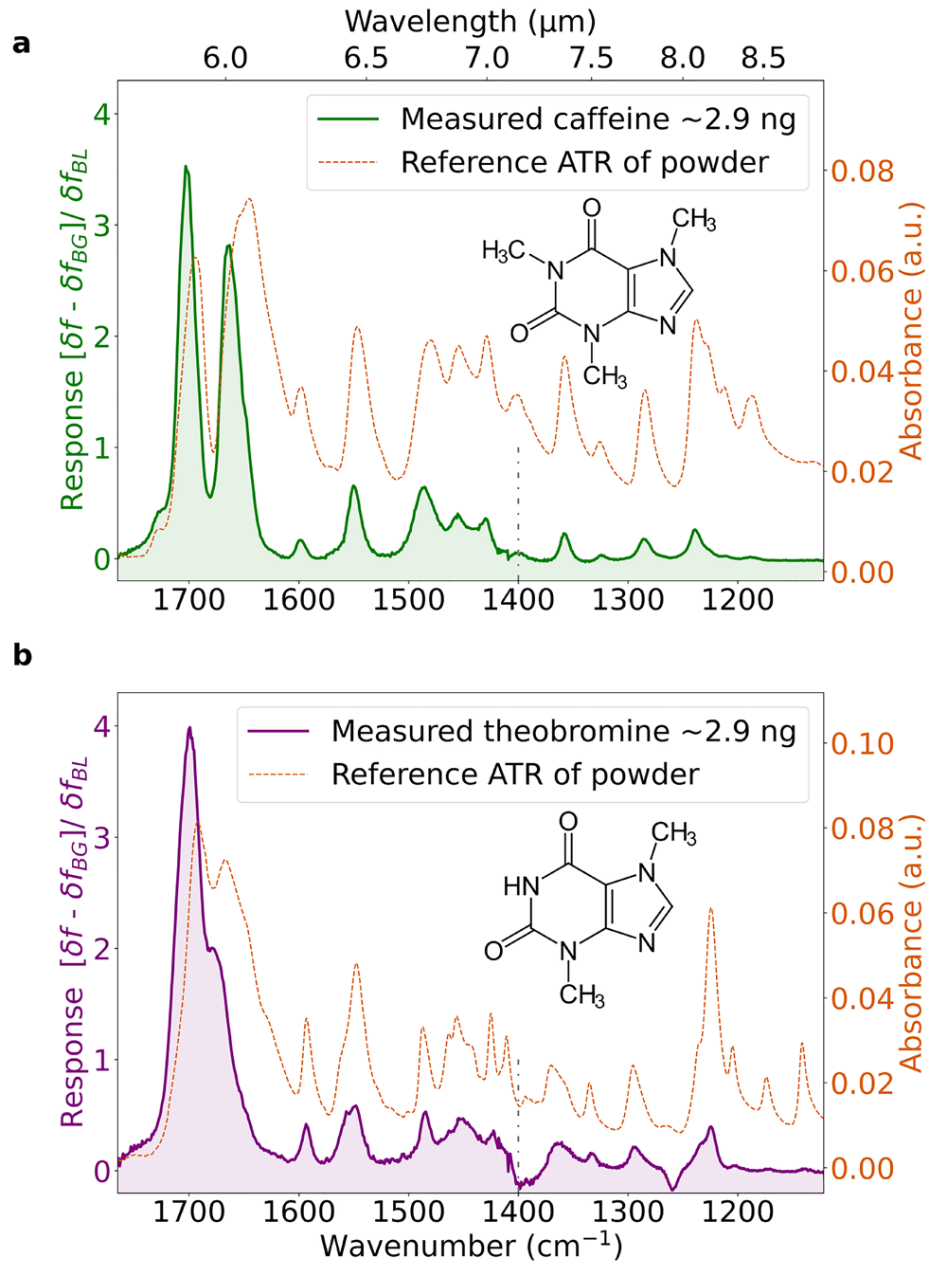
NEMS-IR spectra for (a) caffeine and (b) theobromine sampled on a 1000 μm trampoline resonator and compared to reference spectra obtained from the crystalline powder by ATR-FTIR (PerkinElmer) with 0.5 cm^–1^ resolution.

The NEMS-IR-TD system was first tested with pure chemical compounds. Figure 2 shows the IR spectra obtained for caffeine and theobromine. The response is given as the relative oscillation frequency change *δf* = (*f*_0_ – *f*)/*f*_0_ of the NEMS resonator with base frequency *f*_0_. To compensate for the power variations of the QCL, the spectra were corrected by dividing by the blank spectrum of the bare resonator *δf*_*BL*_ and further subtracting the background spectrum of contaminants (*δf*_*BG*_/*δf*_*BL*_). Comparison to reference spectra recorded on a commercial attenuated-total-reflection (ATR)-FTIR shows nearly identical spectra. In the ATR-FTIR spectra, the absorption band for liquid water is visible at 1640 cm^–1^. In contrast, this water peak is not present in the NEMS-IR-TD spectra, which are acquired in vacuum. The gray dashed line indicates the switching point of the QCL modules at around 1400 cm^–1^ where no power is emitted to the resonator, visible as a slight dip in the IR spectral response.

**Figure 3:**
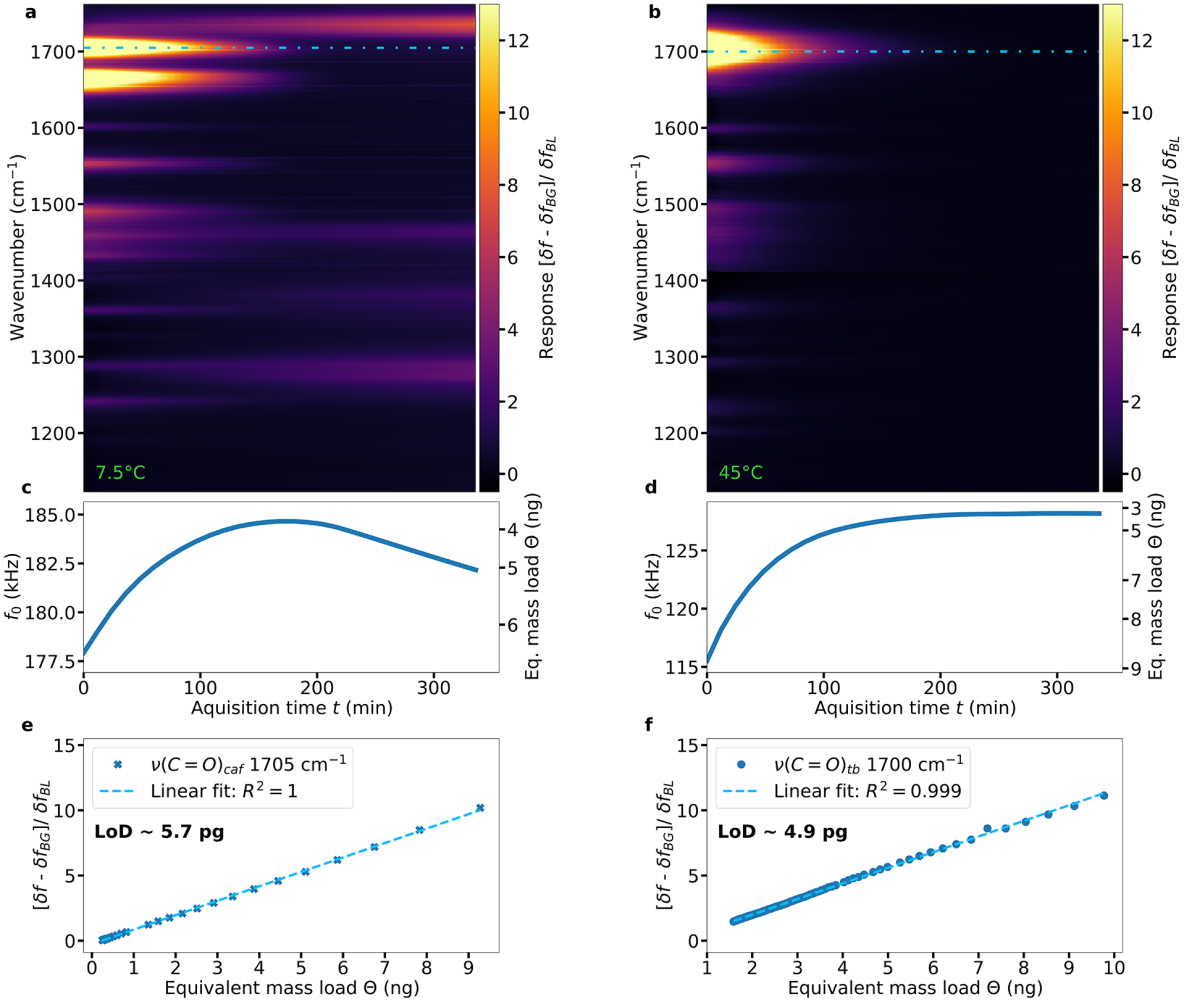
Multidimensional analysis of isothermal desorption in vacuum. Spectro-temporal data for (a) caffeine and (b) theobromine during isothermal desorption from two similar 500 μm trampoline resonators at 7.5 and 45 °C, respectively, and the corresponding mass data of (c) caffeine and (d) theobromine. IR response for (e) caffeine and (f) theobromine as a function of mass load evaluated at 1705 and 1700 cm^–1^, as indicated by the dashed lines in (a) and (b), respectively. For a direct comparison, the LoD analysis that was performed was different from those (a–d) from a single 1000 μm trampoline resonator.

Due to their semivolatility in vacuum, both caffeine and theobromine undergo sublimation and desorb from the NEMS resonator over time. The NEMS-IR-TD setup allows for the simultaneous monitoring of the process by a combination of photothermal IR spectroscopy, shown in Figure 3(a,b), and thermogravimetric analysis, shown in Figure 3(c,d). Assuming an approximately homogeneous mass distribution on the resonator surface, the additional loaded mass of caffeine and theobromine can be calculated from the recorded resonance frequency^34^ (eq 1). Due to the chip being cooled below room temperature for the caffeine measurements, residual contaminants present in the measurement chamber start to adsorb on the resonators’ surface while caffeine is desorbing. This process has two effects: a drop in frequency due to additional mass and the appearance of new peaks in the heat map. As a result, the resonance frequency starts to decrease again after reaching a peak, as shown in Figure 3(c). These new spectral peaks appearing at 1730, 1460, 1380, and 1278 cm^–1^ can be assigned to typical carbonyl, alkane, benzene, and ester hydrocarbon groups, respectively.^35,36^ Thus, in addition to the multidimensional analysis of the desorption process, this technique has the capability to identify trace contaminants. In contrast to the low-temperature and fast desorption of caffeine, theobromine was heated to a much higher temperature in order to trigger desorption. At these higher temperatures, no adsorption of residual contaminants was observed. This can be seen in Figure 3(d) by a constant rise in the resonance frequency up to a plateau where all theobromine has been fully desorbed. From these desorption experiments, the limit of detection (LoD) values for caffeine and theobromine can be calculated by combining the spectral and mass data. The responses of the strongest spectral peak for caffeine ν(C=O)_*caf*_ at 1705 cm^–1^ and theobromine ν(C=O)_*tb*_ at 1700 cm^–1^ were used for the determination of the LoD as indicated by the blue dashed lines in Figure 3(a,b). As seen in Figure 3(e,f), both analytes have a linear response over the entire mass range studied, which spans 3 orders of magnitude. Combining the noise level from the standard deviation σ obtained from five subsequent sweeps with the fitted linear response *R*, one can derive the limit of detection LoD = 3σ/*R* ≈ 5.7 and 4.9 pg (∼30 fmol) for caffeine and theobromine, respectively. In terms of absolute mass collected on the sensor surface, this detection limit is 1 to 2 orders of magnitude lower than that of direct deposition GC-FTIR techniques using cryogenically cooled IR detectors.^10,20^

The additional analysis of the covalently bonded and nonvolatile trimethylsilane monolayer on the silicon nitride NEMS resonators yielded a LoD of 1 ag/μm^2^, which corresponds to approximately 13 zmol/μm^2^ (Supporting Information). This LoD approaches the single-molecule regime that has been achieved with visible light on similar silicon nitride NEMS resonators.^37^ It is estimated that the LoD can be improved by at least 1 to 2 orders of magnitude by (i) reducing the tensile stress of the silicon nitride resonators from 100 MPa down to 1 MPa^37,38^ and (ii) by implementing a different readout method that does not require metal electrodes that pass over the resonator, which deteriorate the photothermal responsivity.^34^

### Desorption Dynamics

**Figure 4:**
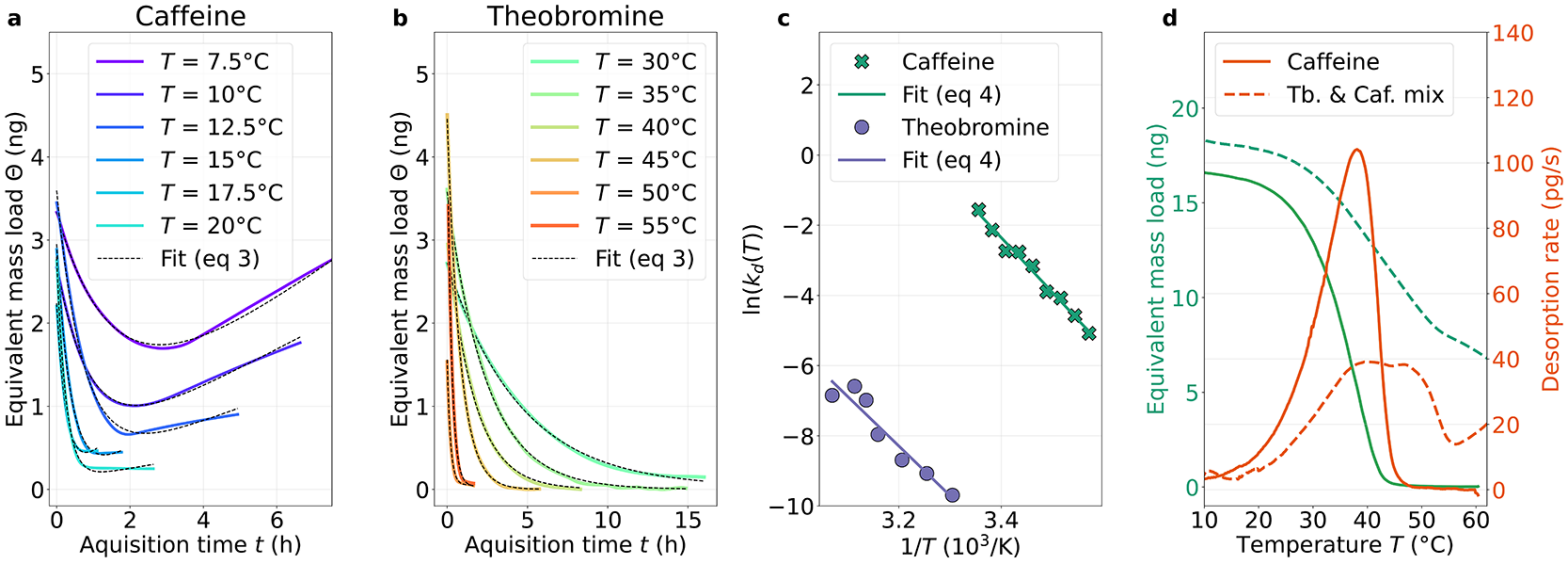
Isothermal and dynamic thermogravimetric analysis (TGA). Isothermal desorption at various temperatures for (a) caffeine and (b) theobromine with an initial mass load of ∼4.5 ng. The mass load data were fitted with the first-order desorption model (eq 3). (c) Corresponding Arrhenius plots for caffeine and theobromine fitted by eq 4 with resulting activation energies and frequency factors of *E*_*d*_ = 130(6) kJ mol^–1^ and ν_*d*_ = 1.14 × 10^22^ s^–1^ for caffeine and *E*_*d*_ = 118(15) kJ mol^–1^ and ν_*d*_ = 1.12 × 10^16^ s^–1^ for theobromine. (d) Dynamic thermogravimetric spectrum for a temperature ramp of 0.1 K s^–1^ for caffeine and a binary mixture of caffeine and theobromine.

The dynamic of the desorption process is studied with NEMS-IR-TD by isothermal and dynamic thermogravimetric analysis (TGA) as presented in Figure 4. Assuming a single desorption process, the desorption rate *r*(*t*, *T*) as a function of time *t* and temperature *T* can be described by the Arrhenius equation^39,40^
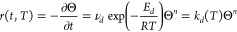
2with the gas constant *R*, the activation energy of desorption *E*_*d*_, the reaction rate constant *k*_*d*_, and the relative surface coverage Θ^*n*^ for a desorption kinetic order *n*. Assuming no dissociation or recombination for the desorption processes observed, the surface coverage/mass loss over time can be described by first-order kinetics (*n* = 1)
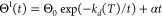
3with the assigning of the initial mass load as Θ_0_ and a linear condensation correction factor α. In Figure 4(a,b), the isothermal desorption at various temperatures is evaluated for both caffeine and theobromine. As seen in Figure 4(a,b), the first-degree desorption model is a good approximation for describing the desorption process. The corresponding sample mass data are fitted with eq 3 to extract the temperature-specific desorption rate constants. Plotting the resulting desorption rate constants *k*_*d*_ versus the temperature according to
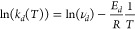
4allows the extraction of the analyte-specific activation energy of desorption *E*_*d*_ and the pre-exponential factor ν_*d*_, as shown in Figure 4(c). The values obtained for *E*_*d*_ are comparable to the sublimation enthalpy values for caffeine and other xanthins measured in former studies (106 to 132 kJ mol^–1^).^41^

In addition to the isothermal TGA, the desorption process can be further studied with dynamic TGA in which the temperature is increased at a constant rate. Here, this is achieved by a programmed stepwise increase of the set temperature at a rate of 0.1 °C s^–1^ (Supporting Information). The data in Figure 4(d) present the desorption spectrum of caffeine and a binary mixture of caffeine and theobromine. The shape of the desorption rate peak of caffeine looks as expected for a desorption process following first-order kinetics.^42^ The desorption rate of the mixture shows two peaks, whereas the second peak is not fully developed at the maximum temperature achievable with the thermoelectric element and just starts to rise. The peak at around 40 °C is due to the selective desorption of the more volatile caffeine. Clearly, the peak shape and amplitude are altered by mixing caffeine with theobromine. The desorption process becomes more complex when multiple molecules are involved. Similar alterations of desorption profiles have also been observed for other binary mixtures.^43^ The dynamic TGA data show the capability of the NEMS-IR-TD system to separate/extract single components out of a mixed sample by thermal desorption. Dynamic TGA provides valuable insight into the composition of a complex sample and can help elucidate information such as the number of components in a mixture as well as the chemical composition of these components when used in conjunction with IR. Due to their similarity in chemical structure, theobromine and caffeine exhibit similar IR spectra (Figure 2). A major difference can be seen at higher wavenumbers, where theobromine contains only a single broad ν(C = O)_*tb*_ absorption peak at 1700 cm^–1^ compared with the double peak of caffeine. As a result, there is significant overlap when analyzing the spectrum of a mixture of caffeine and theobromine. However, thanks to their distinct volatility and desorption rates, the two spectrally similar compounds can easily be separated physically for the analysis.

### NEMS-IR-TD with *In Situ* Sample Separation

**Figure 5:**
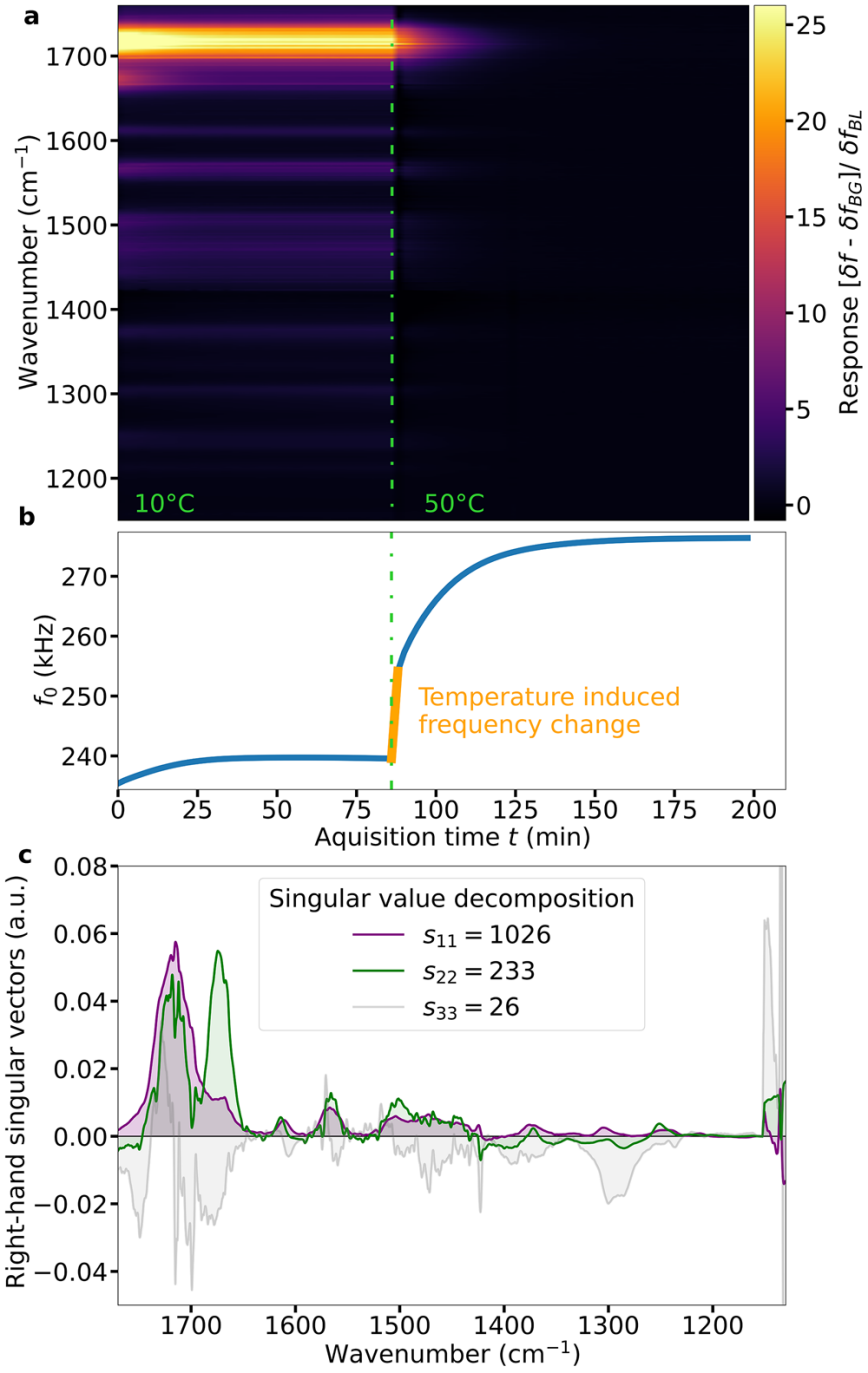
Isothermal separation and single-value decomposition. (a) Spectro-temporal NEMS-IR data of a binary mixture of caffeine and theobromine and (b) corresponding NEMS-TGA data. The green dashed line indicates the temperature change from 10 to 50 °C. (c) Separated spectra at 10 °C from the first three right-hand singular vectors with singular values from SVD.

In addition to dynamic TGA, with NEMS-IR-TD, the desorption dynamics of complex samples can be studied by combining isothermal desorption and continuous chemical analysis with IR spectroscopy. Figure 5(a) presents the spectro-temporal heat map of a binary mixture of caffeine and theobromine recorded at two fixed temperatures. The corresponding mass data are shown in Figure 5(b), proportional to the change in resonance frequency of the NEMS resonator. The large frequency step, when the temperature is increased from 10 to 50 °C, is due to the temperature responsivity of the resonator. At 10 °C, the IR and the mass data indicate the total desorption of caffeine over time. The remaining theobromine can also be completely desorbed when the temperature is increased to 50 °C.

Unraveling the intrinsic spectral and dynamical features of mixtures requires an analysis that can reduce the dimensionality of the data into a few relevant, distinguishable components associated with each species. Similar to spectral analysis techniques of transient spectroscopy, we proceed with two informative analysis procedures: singular-value decomposition (SVD) and global analysis, which is informed by the SVD. First, we determine the number of components contributing significantly to the spectra in the data set as a whole by the SVD. The SVD is a nonsubjective factorization that can be performed on any data set where the spectra vary with some independent parameter such as time^26−28,44^ or pH.^45^ In this top-down mathematical procedure, the singular values indicate a component’s strength in the data set, which sharply declines in further components. Those components with lower values contribute less to the overall response and, likewise, have noisy, unstructured right- and left-hand singular vectors. These vectors represent the spectra and their associated dynamic traces, respectively. Based upon the number of components demonstrating structured singular vectors, SVD allows for the choice of a minimum number of components, which sufficiently represent the entire spectro-temporal response. For caffeine and theobromine in a mixture at 10 °C, four components were sufficient. The right-hand singular vectors of the data set for caffeine and theobromine in the mixture at 10 °C are shown in Figure 5(c), with the exception of the fourth structureless component (Supporting Information Figure S8). SVD separates the theobromine-dominant (*s*_11_) and caffeine-dominant (*s*_22_) desorption contributions and properly factors in an inverted spectral feature (*s*_33_), suspected to be condensing contaminants. The singular values indicated in the legend of Figure 5(c) show that, for the duration of the experiment, the primary contribution to the total resonance frequency shift was theobromine. Caffeine contributes less, due to its short lifetime, as does the contaminant, due to the time limitation of the experiment. The associated left-hand singular vectors (Supporting Information Figure S8) can be fitted to estimate the rate of change of each component.

**Figure 6:**
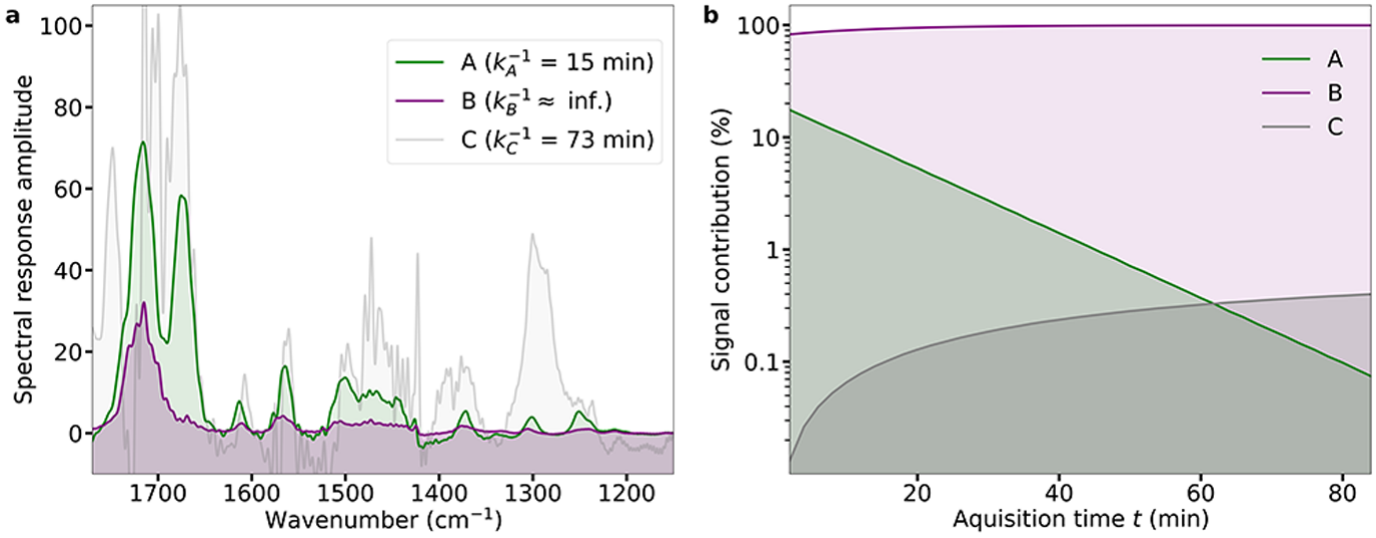
Separation example by global analysis. (a) Spectral response amplitudes (or evolution-associated spectra) from global analysis applying a sequential model to the spectro-temporal data at 10 °C in Figure 5 up to the temperature change at 86 min. (b) Corresponding relative contributions of the spectra in (a) to the total response. The superposition of the outer product of each spectrum in (a) and corresponding traces in (b) reproduce a fit to the spectro-temporal data in Figure 5.

By the SVD, we obtain the number of primary contributing components and their general spectral form, and this informs our second method of analysis, global analysis, which is a multidimensional fitting procedure. In this bottom-up approach, shown in Figure 6, we can distinguish trends in these spectro-temporal maps, where first-order, exponential trends are fit across the whole spectrum. This method is widely used to identify decaying transient species in ultrafast spectroscopy only after performing the SVD,^27,29^ whereas instead of the analysis of the transient dynamics of populated electronic excited states the evolution of transitory resonance frequency shifts during the desorption or adsorption of IR-active species is evaluated. The *Ultrafast Spectroscopy Modeling Toolbox* from von Thor and colleagues^27^ was chosen to showcase the potential of NEMS-IR-TD upon full utilization of the information the method provides. The number of components required for the analysis of the caffeine/theobromine mixture at 10 °C was informed by dynamic TGA and SVD. These so-called evolution-associated spectra reveal three distinct spectral trends arising in succession, beginning with caffeine (residues shown in Supporting Information Figure S9). The first two components, decaying at rates of *k*_*A*_^–1^ = 15 min and *k*_*B*_^–1^ = inf., are primarily characteristic spectral features of caffeine (A) and theobromine (B). The final, slowly rising component, with the same peaks as observed in Figure 3(a) at later times, identifies the spectrum of volatile hydrocarbon condensation (C), which monotonically rises throughout the time of the experiment.

As the SVD reveals distinct components according to their contribution to the NEMS-IR-TD data, global analysis yields the evolution of distinct and identifiable spectral signatures according to a sequential model. However, the exact spectra of the individual adsorbing species and their rates can be obtained by target analysis if the sorption reaction scheme or model is known. Such an attempt to obtain the so-called species-associated spectra is shown in Supporting Information Figure S9 according to a mixture of sequential and parallel processes. Therefore, NEMS-IR-TD data allow for a full range of spectral analyses for species separation on the femtomolar scale.

## Discussion and Conclusions

With NEMS-IR-TD, we present a novel method for the spectral and thermogravimetric analysis of single and mixed compounds with femtomole sensitivity. Testing this method with two xanthins exhibiting similar IR spectra but distinct vapor pressures showed the capability of *in situ* separation by isothermal desorption as well as by spectral analyses recorded with a single device. Due to the rising capabilities of chemometrics based on dedicated algorithms up to artificial intelligence,^1,2^ we expect that mixtures with similar vapor pressures can also be identified. Besides the spectral deconvolution, one can further adjust the temperature to enlarge the acquisition/desorption time and thereby the number of data points in which trends can be picked up by global analysis. However, further studies with a focus set on the separation limitations still need to be conducted.

The sampling method used is optimal for capturing airborne nanoparticles such as environmental aerosols with a size down to about 20 nm.^32^ Here, this sampling method is applied to capture molecules initially solvated in solution. The resulting airborne particle size is correlated to the concentration of the sample solution. Hence, a very low sample concentration results in an aerosol particle size which is too small, limiting the sampling efficiency (Supporting Information). This limit could be overcome by changing the deposition method to low-pressure direct deposition,^10^ electrospray and molecular beam deposition,^46^ or selective capture by surface functionalization, which could allow for trace analysis with single-molecule sensitivity as the ultimate limit.^37^

The temperature range of the setup was relatively small. Thus, the maximum temperature was not high enough to complete the temperature-programmed desorption of theobromine, which has a relatively low vapor pressure. The range of the temperature control could readily be improved by using thermoelectric stacks. An enhanced temperature range would increase the range of samples that can be analyzed. Lower temperatures could allow the capture and retention of volatile compounds, such as volatile organic compounds (VOCs),^47^ while the ability to reach higher temperatures could allow the desorption of compounds with lower volatility.

Singular value decomposition and global analysis of the time-dependent information provided by NEMS-IR-TD readily yield species-specific information from the isothermal desorption processes (Figures 5 and 6), whereby identifiable spectral signatures of individual species are extracted from the convoluted frequency response to IR absorption over time. Their spectral signatures are temporally separated by their interaction with the adsorbent and other adsorbates. This opens the possibility of tailoring the surface of the NEMS resonator for selective species adsorption as well as surface passivation studies. In this context, it was possible to detect one of the O–Si–(CH_3_)_3_ vibrational modes from the nonvolatile surface passivation (trimethylsilane) with a high signal-to-noise ratio, showing that NEMS-IR-TD is well suited for the study of single monolayers and submonolayers (Supporting Information Figure S6).

In this work, theobromine desorbed at a much slower rate than caffeine, individually and in a mixture, allowing its spectral features to be differentiated from that of caffeine’s. In view of this, dynamic TGA and SVD alone demonstrate vast utility. This combination provides the characteristic spectral signatures and the number of chemical species present, and an inversion of the spectrum distinguishes adsorbing species from the desorbing species. Additionally, the SVD reveals the relative contributions of each component to the total frequency shift. Once a sufficient number of components are determined by SVD, a more intuitive analysis, such as global analysis and target analysis, can be applied. First-order global analysis in Figure 6 reveals the evolution of three distinct spectral signatures and their associated dynamic trends. The analysis is even able to distinguish subspecies spectra of caffeine isomers and how their contribution changes with temperature (Supporting Information Figure S10). To our knowledge, this is the first demonstration of utilizing a powerful and highly versatile tool for the analysis of disparate, simultaneously desorbing and adsorbing species. This substantiates the potential for future developments using global and target analyses, including higher-order^28^ and even deep-learning analyses,^48^ tailored to specific NEMS-IR-TD applications. Therefore, we expect that this technique will find a wide range of applications for the physicochemical characterization of complex samples in various fields from environmental analysis to life sciences.

## Supporting Information Available

In-depth explanations of the optimized resonator design, sampling setup, spectral analysis and its deconvolution down to the identification of subspecies; further the detection of the passivation layer (PDF)
