# Supplementary material for: Nanoelectromechanical Infrared Spectroscopy with In Situ Separation by Thermal Desorption: NEMS-IR-TD
Source: ACS Sens. 2023 Apr 17;8(4):1462–70. doi: 10.1021/acssensors.2c02435 (PMC10152476; doi:10.1021/acssensors.2c02435)
Supplement: Supplementary file 1 — se2c02435_si_002.pdf [file se2c02435_si_002.pdf]

Supporting Information: Nanoelectromechanical infrared spectroscopy with *in situ* separation by thermal desorption: NEMS-IR-TD

Niklas Luhmann,[*,†] Robert G. West,[†] Josiane P. Lafleur,[‡] and Silvan Schmid[*,†]

†*Institute of Sensor and Actuator Systems, TU Wien, Gusshausstrasse 27-29, 1040 Vienna, Austria.*

‡*Invisible-Light Labs GmbH, Taubstummengasse 11, 1040 Vienna, Austria.*

E-mail: niklas.luhmann@tuwien.ac.at; silvan.schmid@tuwien.ac.at

Resonator design and stress distribution

For this study, the resonator design was optimized to compromise between thermal isolation for enhanced response and ensure robustness for fabrication yield and aerosol sampling. Therefore several resonator designs in a trampoline shape with different curvatures were fabricated and simulated with COMSOL Multiphysics and tested in the setup beforehand. One key feature to ensure a more robust and smooth stress distribution throughout the entire resonator was the implementation of a round clamping design - following previous studies [1]. As can be observed from the simulation, presented in Fig. S1, a round clamping with a virtual radius of 20 µm shows a much lower peak stress when compared to a regular/direct clamping. Further, the round clamping provides a more smooth stress distribution along the tethers while still maintaining the desired stress-reduction of the central area to increase the

thermal response. From this analysis and several test measurements, optimal performance was achieved by trampoline resonators with a lateral size of 500 µm and 1000 µm, and a broader curvature compared to highly optimized geometries for thermal response[2].

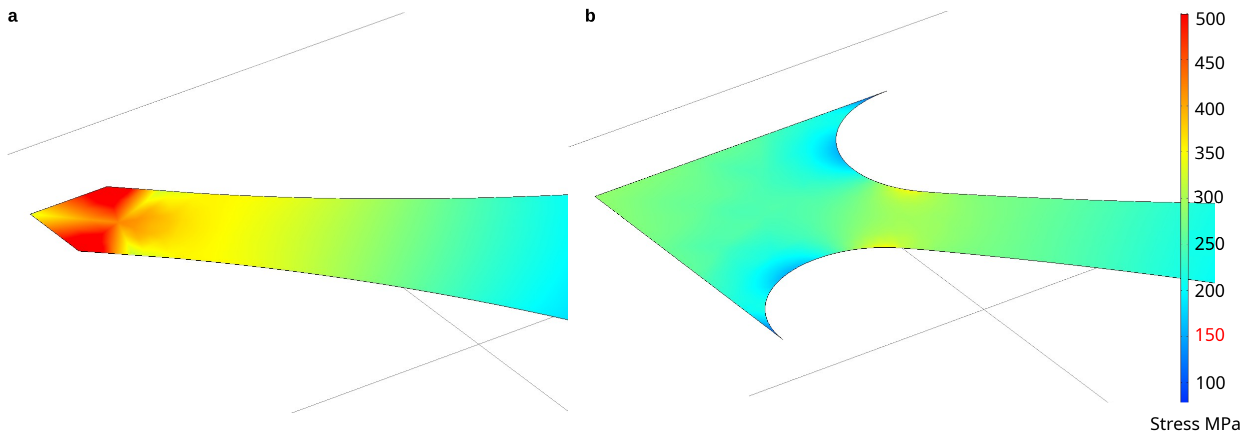

Figure S1: Simulated stress distribution of the trampoline clamping area with an initial tensile stress of 150 MPa. Changing the clamping from a direct connection (a) to a round edge (b) with a virtual radius of 20 µm significantly reduces peak forces in the clamping area.

Impact of aerosol based sampling method on efficiency and homogeneity

For the analyte deposition a in-house aerosol sampling similar to an effluent elimination systems in LC-FTIR was used. The liquid analyte is pulled in via a 20 µL min^{-1} self-aspiration capillary by means of the *Venturi* effect from a pneumatic jet nebulizer (ESI MicroFlow PFA-ST), which generates a fine spray into a cyclonic spray chamber (MEINHARD-ESI ML148030). Due their inertia, the spray chamber acts as a cutoff-filter for larger droplets, while smaller droplets get reduced to a dry particulate aerosol when passing through diffusion dryer (TOPAS DDU 570/L). In a final step, the residual dried aerosol is flushed through the orifice and perforation of the resonator chip. In order to prevent the aerosol to bypass the resonator, the chip is sealed in a homemade teflon chamber between two NBR O-rings, pressed down by a 3D printed magnet bride (see Fig. S3(a)). A comparison of the success-

fully sampled mass with the available total mass from the analyte showed a significant lack and variation of the sampling efficiency. This can be evaluated by comparing the total mass of the analyte in liquid with the resulting loaded mass on the resonator.

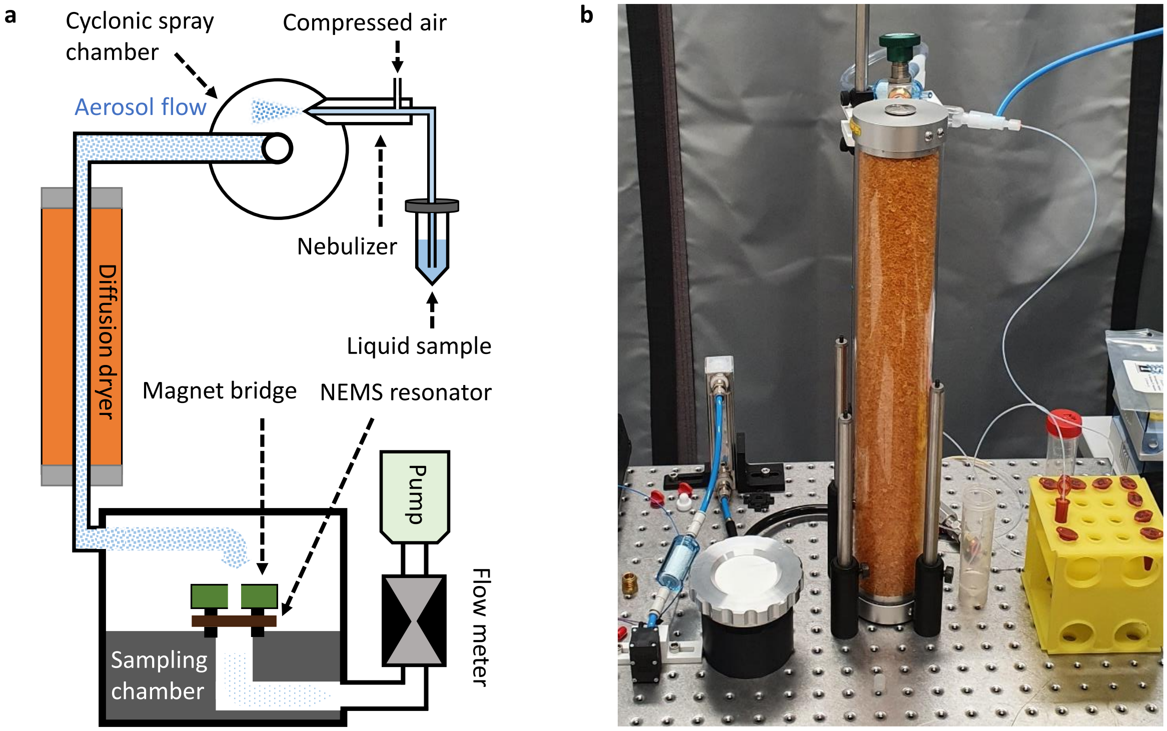

Figure S2: (a) Schematic draft and (b) picture of the homemade aerosol sampling setup comprising mainly a jet-nebulizer, diffusion dryer, sampling chamber and flow adjustable pump. The liquid analyte is introduced by a 20 µL self-aspiration capillary and the *Venturi* effect of the nebulizer. The resulting droplets are passing a cyclonic spray chamber acting as a cut-off filter. Subsequently, the droplets are dried to a particulate aerosol and sampled by interial impaction when flushing through the perforation of the chip.

From a measurement using step-wise increasing analyte volumes of 30 µL, 60 µL, 90 µL and 120 µL with a fixed concentration of 60 µg mL^{-1} and the resulting measured mass-load on the resonator; the sampling efficiency is in the range of 0.44 % to 0.55 %. A huge mass loss in the system can be linked to the cyclonic spray chamber where the majority of the analyte gets lost due to larger droplets. This can be significantly improved by an optimized spray chamber made for ICPMS, which is currently tested in our facilities. Furthermore, the stress optimized trampoline resonators have openings on each side, which increases the flow-through area and probability for particles passing with the streamline without any

interaction to the substrate. This issue can be addressed by changing the resonator design back to a perforated membrane. However, this would lead to a reduced thermal response and sensitivity. Alternatively, the sampling scheme can generally be changed to methods based micro-dispensers, electrospray or e.g. by a tailored surface functionalization.

Besides the discussed lack in sampling efficiency, the design of the homemade sampling chamber has caused the deposition of partly in-homogeneous layers. Figure S3 (a) shows a picture of the inner part of the chamber and a micro-graph of the trampoline resonator - sampled with ≈ 38 ng caffeine. The reduced orifice of the magnet bridge which was used to fixate and seal the chip for sampling is causing a vortex of the aerosol flow down to the resonator. Thereby, some areas are covered by a more condensed layer of the analyte while others are empty and show the bare silicon nitride of the resonator. This issue can be solved by modifying the sampling chamber and specifically improve the aerosol flow over the chip when using aerosol based sampling.

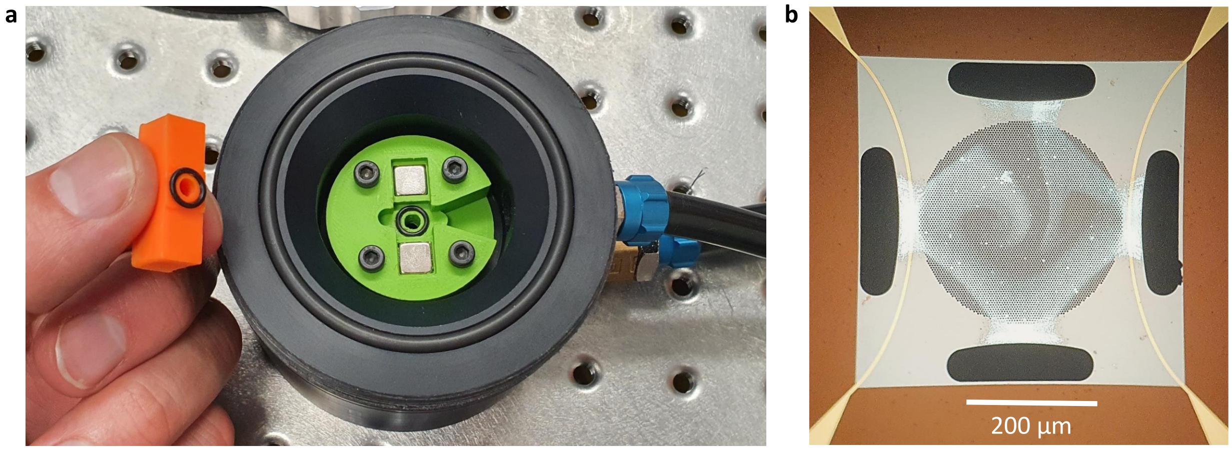

Figure S3: Picture of sampling chamber (a) and micro-graph of a sampled chip (b). Due to the orifice of the used magnet bridge the aerosol formed a vortex over the resonator. This has caused an in-homogeneous distribution of mass during sampling instead of defined monolayers.

Simulation and calibration measurement of chip temperature

A key component of the thermal desorption based separation is a precise control of the resonators temperature. Here, a thermoelectric element (1MD06-015-15H - TEC Microsystems) is thermally coupled to a 6 mm thick copperblock, acting as thermal sink. The set temperature for the PID is measured with a thermoresistor (PT1000) placed in the upper side of the copper-block (see manuscript Fig. 1). Since the resonator chip is placed on top and not enhanced thermally connected by conducting paste, the resonator temperature differs. Therefore, a dummy-chip was equipped with a thermoresistor (PT500) which is connected via the spring-loaded contact bridge to acquire a calibration curve. Figure S4 (b) shows a picture of the dummy-chip in the chamber and Fig. S4 (a) the measured temperature over the applicable range compared to the PID set points.

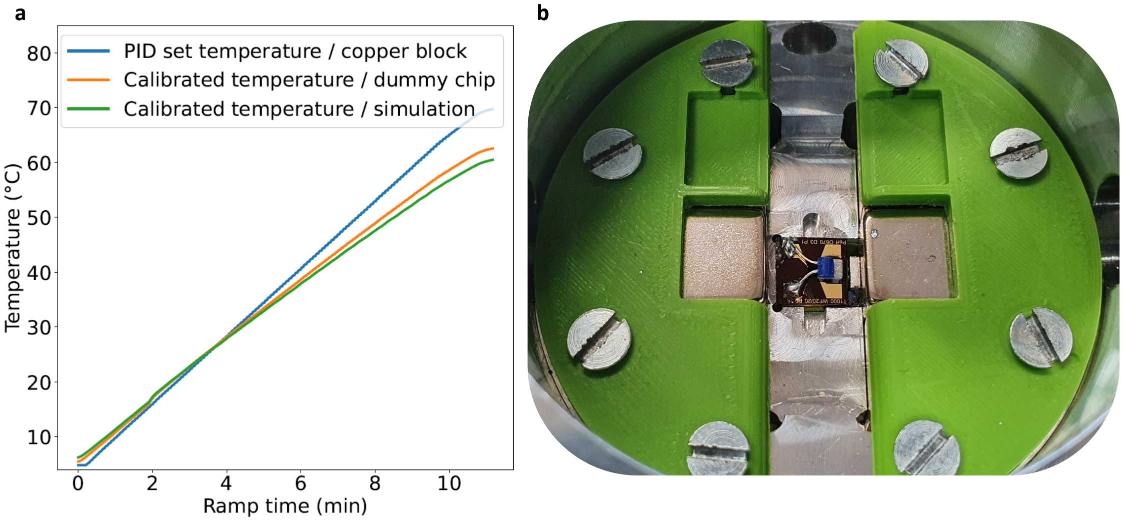

Figure S4: Calibration of resonator temperature. (a) set-point and measured temperature of the thermoelectric element compared to a dummy-chip equipped with a PT500 thermistor. (b) the dummy chip is connected via the spring-loaded contacts to a second port of the temperature controller. Due to the decreased thermal conductance in vacuum, the chip can not be heated and cooled to the pre-set temperature of the copper block beneath.

In addition to the calibration of the chip temperature, also the low thermal conductance of the silicon nitride needs to be taken into account. Previous studies have shown, that the reduced thermal conductance of suspended silicon nitride and influence by ambi-

ent radiation can significantly alter the resonator temperature[3]. This effect was estimated by finite-element-method simulations using COMSOL Multiphysiscs 5.4. Figure S5 shows the simulated temperature distribution of a quarter trampoline resonator with a set frame temperature of (a), 5 °C and (b), 55 °C assuming a surface-to-ambient radiation with an emissivity of $\varepsilon = 0.05$[3]. Due to the 100 times higher thermal conductance of gold, the frame temperature remains almost constant along the electrodes but significantly drops towards the resonators' center. In order to achieve an estimate of the resonator and analyte temperature, respectively, the average of the temperature along a 150 µm diagonal line from the center was evaluated. For an estimation of the temperature during a static and dynamic TGA run, this average was calculated from a parameter sweep using the previous calibrated chip temperature values. The result is plotted in Fig. S4(a).

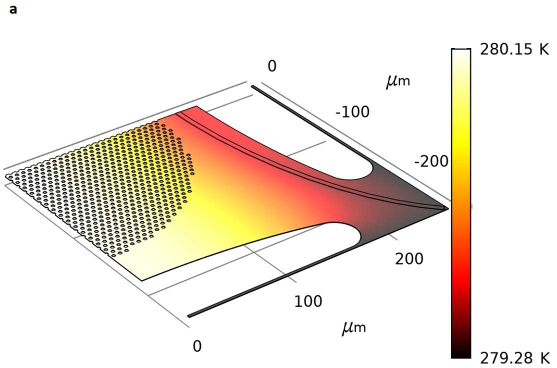

Figure S5: Finite element method simulation of temperature distribution on the resonator. (a) Distribution for a chip-frame temperature of 5 °C, (b) for a set chip-frame temperature of 55 °C. Due the very low thermal conductance of the silicon nitride with $3\,\mathrm{W\,K^{-1}\,m}$, the average temperature of the resonator can differ up to $\Delta \approx 3$ °C from the frame.

Detection of passivation layer

In the course of this study the resonators where passivated with a solution of Trimethylchlorsilane (TMCS) following a protocol from M. Szkop et al.[4]. As a result, a monolayer of oxigen bonded Trimethylsilan (TMS) (O-Si-$(CH_3)_3$) is formed on the native oxide surface of the resonator. Figure S6(a) shows, that the Si-$(CH_3)_3$ exhibits a strong IR-active bending mode at $1263\,\mathrm{cm}^{-1}$ (also when bonded as passivation layer see[5,6]). Comparing the thermal response and resulting background spectrum of a regular trampoline resonator with a passivated resonator, as depicted in Fig. S6(b), this mode can been readily detected also by NEMS-IR. In order get an estimate of the detection limit of NEMS-IR for such a monolayer, one can assess the number of TMS groups involved to the response measured. Previous studies on the packing density for TMS passivation obtained an average steric limit of 2.41 TMS groups per nm^2[7]. Considering the molecular mass of TMS, this resembles a surface density of approximately $0.3\,\mathrm{fg}\,\mu\mathrm{m}^{-2}$. Finally, one can include the signal-to-noise ratio for the measured mode $\delta(\mathrm{Si\text{-}(CH_3)})$, shown in Figure S6(c). With a noise level of five subsequent scans, we obtain a ratio of about SNR $\approx$ 337. Therefore, the detection limit of NEMS-IR for this passivation monolayer is in the range of $0.89\,\mathrm{ag}\,\mu\mathrm{m}^{-2}$.

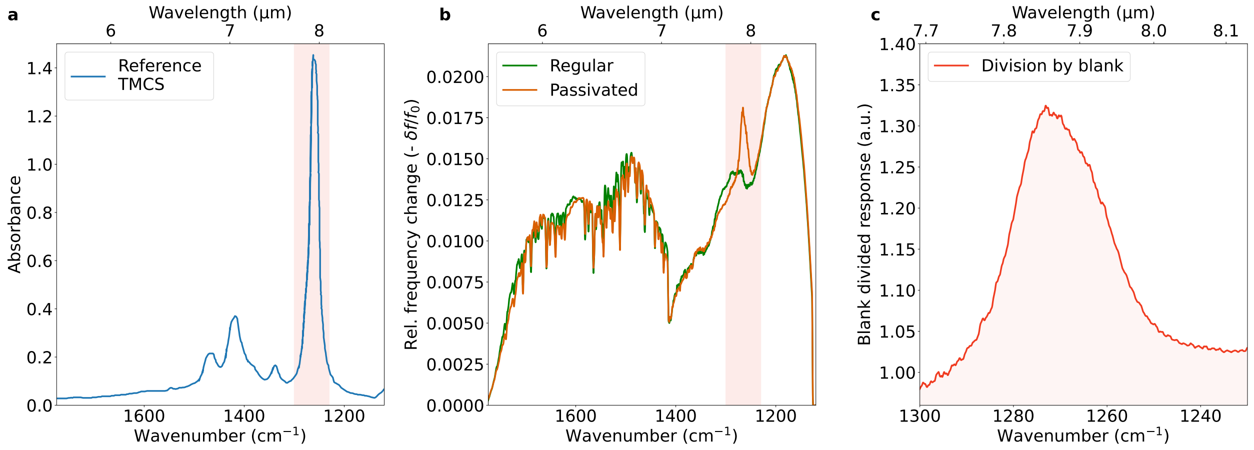

Figure S6: NEMS-IR detection of the passivation monolayer. (a), Reference FT-IR spectrum of TMCS showing the strong deformation mode of $\delta(CH_3)$ at $1263\,\mathrm{cm}^{-1}$ which can be identified in background spectrum of the passivated resonator in (b). (c) Shows the resulting response of the vibrational mode when dividing through the background of a regular unpassivated resonator of the same dimensions.

Spectral shifts by misaligned timing

Due to the communication between laser, lockin and computer, a timing related shift occurred. This has led to a constant offset of the emission wavenumber to the polled resonance frequency in the acquired spectrum. Figure S7 shows this effect for different sweep rates compared to a step-mode measurement in the range of the passivation response δ(Si-(CH_3)). Thus, for all spectra recorded with a rate of $25\,\mathrm{cm^{-1}\,s}$, a wavenumber correction factor of $-11.68\,\mathrm{cm^{-1}}$ was applied. Several comparisons between literature values and the measured IR modes of caffeine, theobromine, hydro-carbon bonds and the response by the passivation layer showed, that this correction factor seemed valid.

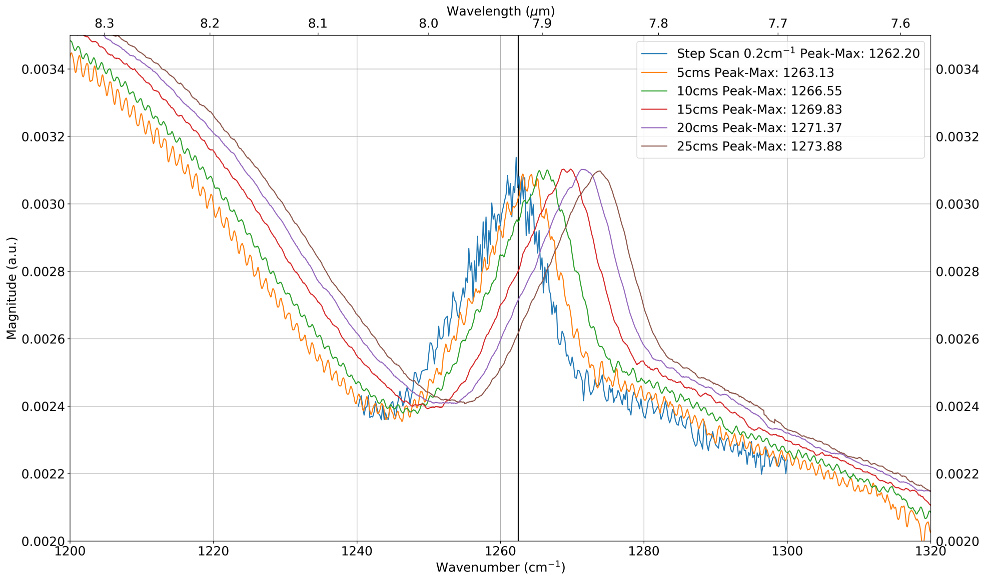

Figure S7: Spectral shift due to misaligned timing between laser emission and recording of the resonance frequency. The absolute shift was evaluated around the passivation layer response and demonstrated for different sweep rates compared to step-scan measurement.

Application and data processing for thermogravimetric analysis

In order to perform a dynamic TGA measurement a temperature ramp was programmed using the PID control software of the thermoelectric element. This was done by a step-wise incremental increase of 0.5 °C of the temperature set-point every 5 s and a reduced propor-

tionality factor of the PID to get a smooth regulation. The result was an approximately linear temperature ramp with a rate of 0.1 °C s^{-1} ranging from 5 °C to 70 °C. Due to the reduced thermal coupling of the chip to the copper block and further considering the low thermal conductance of the resonator itself, the actual temperature at the analyte needed to be corrected. The frame temperature of the chip was therefore measured by a dummy-chip equipped with a thermistor to record a calibration curve. The resonators' average temperature was then calculated by finite element method simulations including the calibrated frame temperatures (see Supplementary Fig. S4&S5). In order to compensate the change of the resonance frequency due to the temperature, a blank temperature ramp of the empty chip was recorded after the analyte was fully desorbed. This was ensured by measuring an NEMS-IR spectrum before and after the temperature ramp. The mass-load was then calculated with Eq. 1 (main manuscript), while using $f_0(T)$ from the blank ramp. In a final step, the desorption rate was calculated from the mass-loss between consecutive temperature steps of 0.1 °C.

Spectral Analyses

Three types of analyses were applied to the spectro-temporal maps obtained in these NEMS-IR-TD studies: singular value decomposition, separating the data into components by their contribution strength; first-order global analysis, separating components by their sequential contribution; and target analysis, in an attempt to separate components according to a prescribed reaction scheme.

Singular Value Decomposition

Singular Value decomposition (SVD) for the caffeine/theobromine mixture at 10 °C demonstrates that there are three spectrally-distinct components associated with theobromine, caffeine, and condensate contributions (Fig.S8). However, a fourth component, an infinitely

long lifetime component was required to fit the data well. Nonetheless, this infinite component, not shown in the paper, is generally flat across the spectrum and noisy, following the trend of mass loss throughout the experiment.

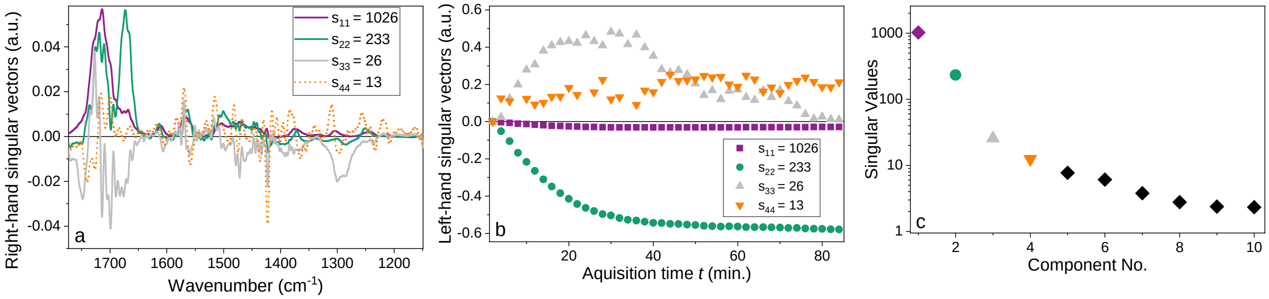

Figure S8: The full right-hand (a) and left-hand (b) singular vectors along with their associated singular values (c) for the caffeine and theobromine mixture data at 10 °C.

Singular value decomposition (SVD), performed with Matlab's built-in `svd` function, is applied as a rank reduction procedure, whereby the heat-map (Fig. 3(a&b) in the main text) is essentially factored into a finite number of orthornormal basis vectors, named right-hand singular vectors ($\boldsymbol{V}$), whose columns represent the constituent spectra, and left-hand singular vectors ($\boldsymbol{U}$), whose columns represent the time traces[8–10]. The data map can be sufficiently represented, therefore, by the factorization

$$\boldsymbol{M} = \boldsymbol{U}\boldsymbol{\Sigma}\boldsymbol{V}^T = \sum_{i=1}^{r} s_{ii}\mathbf{u_i}\mathbf{v_i}^T, \tag{1}$$

where the diagonal matrix $\boldsymbol{\Sigma}$ contains the set of singular values $\{s_{11}, s_{22}, ...\}$ weighting the contribution of each component $\mathbf{u_i}\mathbf{v_i}$ composing the heat-map $\boldsymbol{M}$. The sets of left and right-hand singular vectors (Figure S8(a & b)), $\{\mathbf{u_1}...\mathbf{u_r}\}$ and $\{\mathbf{v_1}...\mathbf{v_r}\}$, are the dynamic traces and spectra, respectively, which form the orthonormal bases for the dataset. Comprising the columns of $\boldsymbol{U}$ and $\boldsymbol{V}$, they are the eigenvectors of $\boldsymbol{M}\boldsymbol{M}^T$ and $\boldsymbol{M}^T\boldsymbol{M}$, respectively, and their eigenvalues are the square of their singular values (Figure S8(c)). The value of preforming

SVD on spectro-temporal data is found in reducing the data to a small number r of the most relevant components sufficient to represent the data set. As the singular values decrease with r, so does the prominence of their associated left and right-hand singular vectors in the overall data set. Likewise, the associated spectra that are contributing less, appear more noisy with increasing r. $\boldsymbol{M}$ can, therefore, be the matrix which sufficiently represents the original data matrix with the least amount of components r, and it is of rank r.

Global and Target Analyses

Global and target analyses of the data map was also performed using the *Ultrafast Spectroscopy Modelling Toolbox*[11]. With exception of the corrections made for pulse chirp and the vast difference in time scales and wavelengths typical of ultrafast spectroscopy, there is no difference in the basic utility of this method for an exponential analysis of NEMS-IR-TD data. The SVD was used to inform the minimum number of required components in the global analysis as well as their approximate rate of sorption. The quality of the fit is determined by reduction of the sum of the residuals of a fit to the whole heat-map by a combination of gradient and direct search methods. The toolbox utilizes Matlab's *Patternsearch* algorithm for determining the local minimum in the parameter set composed of initial conditions and rates between states of the system being analysed. Likewise, the connection scheme and initial conditions for these states, labeled "species", was built in Matlab's Open SimBiology desktop (now Open SimBiology Model Builder), also utilized by the toolbox. The analysis extracts time-independent correlations, each with its own characteristic spectra and associated transitory lifetime given by the first-order rate law for each species. Any zero-order reactions will be perceived, therefore, as a component that grows or decays with an infinite lifetime. Just recently, an open-source global and target analysis package for Python language has made spectral analysis of higher order reaction kinetics possible[12]. However, a sequential, first-order model can necessarily describe the system as its spectra evolves from state to state.

By these so-called Evolution Associated Spectra, a reaction connectivity scheme can be informed, even inferred. Though the proportion of concentration of caffeine to theobromine in the analyte mixture was 1:1 in stock solution, the initial populations of the first caffeine-associated state and the theobromine state were set to 1:4 in these analyses. This is because caffeine had time to desorb faster than theobromine during the resonator chip placement, chamber pump down, and temperature stabilization before the measurement began. The exact proportion was chosen to reduce the cross-contamination of spectral features among the resulting spectra. Varying this proportion in the model did not have a significant effect on the lifetimes.

The global analysis overemphasizes the final C component in the spectra, but this is compensated by the small total contribution in the dynamics. Species C behaves more accurately as a condensate in the target model; whereby, the spectrum is inversed as condensation would cause a resonance frequency shift opposed to that of desorbing species A and B. It is a mirror image of species C in the global analysis. Likewise, it is expected that as the caffeine-associated species, A, desorbs more quickly, condensation may replace the surface previously covered by caffeine more quickly according to the target model.

In an attempt to extract the species-associated spectra and dynamics, target analysis was also applied (Figure S9). Instead of a three-state sequential scheme, a more plausible parallel scheme of two species transferring simultaneously to a third dynamic state was used. In more complex reactions, global analysis would, nonetheless, give valuable insight into the evolution of the system but more exact knowledge would be required for a targeted model. Nonetheless, recent advancements in deep learning applied to photochemistry has made it possible to posit reaction schemes with a certain level of confidence based upon such spectro-temporal data[13]. Global and Target analyses, according to the connection schemes in Figure S9(a), produce spectra (Figure S9(b)) that, when weighted by their percent contribution in time (Figure S9c) and added together, form a fit to the whole data map. The global analysis shown here in Figure S9 (solid lines), is a standard means of evaluating how the system

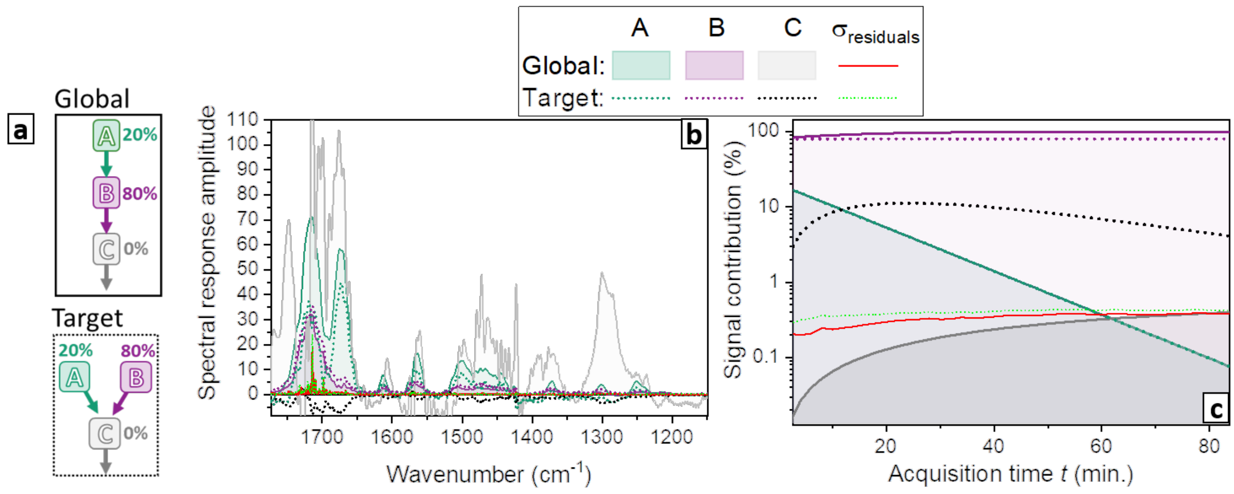

Figure S9: Global and Target analyses of the Caffeine-Theobromine mixture data at 10 °C. (a) Schematics of global and target analyses connection schemes with initial signal contribution per species (A, B, C). (b) The evolution-associated spectra of global (solid lines) and species-associated spectra of target analyses (dashed lines) and the standard deviation of the residuals $\sigma_{residuals}$ over all time. (c) The associated dynamical traces representing the total signal contribution of each component in time and the standard deviation of the residuals $\sigma_{residuals}$, which scale the residuals in (b).

evolves through spectral trends in time. This is why the sequential, global analysis produces the so-called evolution-associated spectra (Figure S9(b)) and results in distinct, identifiable spectral trends, which closely resemble the chemical species involved. The second analysis is a so-called target analysis, whose connection scheme among the species (Figure S9(a)) corresponds to a proposed reactivity scheme. If the connection scheme is accurate to the true reactivity scheme in the experiment, the resulting spectra are the species-associated spectra. Indeed, species A in the target analysis more closely matches the reference ATR spectra of caffeine in Figure 2(a) in the article than the evolution-associated spectra. The dynamic traces of the sequential global and semi-parallel target models naturally differ and are shown in Figure S9(c).

In order to demonstrate the quality of fit, we have calculated the standard deviation of the residuals in the spectra at a given wavenumber (k) by

$$\sigma_{spec}(k) = \sqrt{\frac{SS_{spec}(k)}{N_{timepts} - 2}}, \tag{2}$$

where SS_{spec} is the sum of the squares of the residuals for all time in the fit and $N_{timepts}$ is the number of time points measured.[14] Likewise, the standard deviation of the residuals in the time traces were calculated, subsequently graphed in percent of the total signal,

$$\sigma_{trace}(t) = \sqrt{\frac{SS_{trace}(t)}{N_{wvnum} - 2}}, \tag{3}$$

where SS_{trace} is the sum of the squares of the residuals over the whole spectrum for a particular time (t) in the fit, and N_{wvnum} is the number of points in the spectrum. It is important to note that the residuals shown the time traces (Figure 9(c)) is scaled by the magnitude of the residuals in the spectra (Figure 9(b)) and vice versa.

In global analysis of *caffeine-only* data (Figure S10), more than one component is required to fit the data set. There are multiple interactions on the surface producing simultaneous dynamics. These evolution-associated absorption spectra are capable of distinguishing individual trends in the caffeine data, indicating multiple temperature-dependent subspecies on the resonator. Apparently, for this particular chip with passivated surface, from 12.5 °C to 15 °C, the caffeine species undergo a change in their dynamics and contribution to the resonator frequency response.

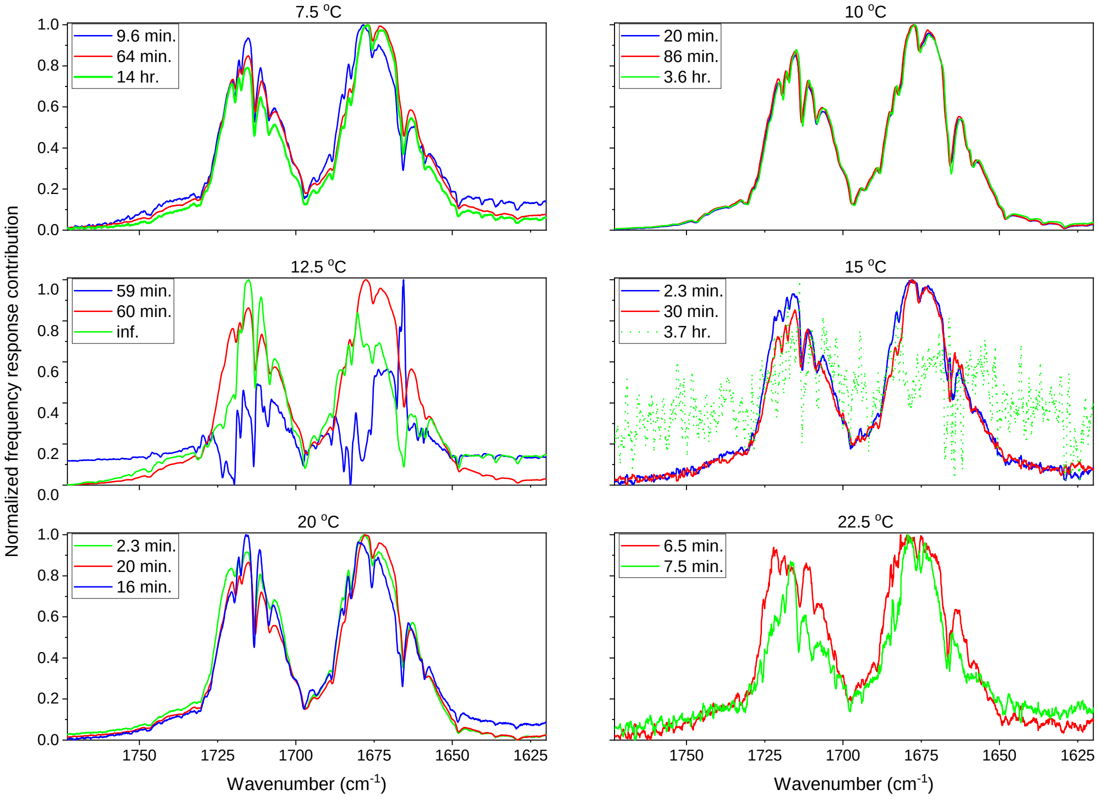

Figure S10: Normalized frequency shift contributions of the first three components of caffeine-only data at various temperatures by global analysis, revealing caffeine subspecies. The first component at 22.5 °C was too fast to be observed.

References

(1) Sadeghi, P.; Tanzer, M.; Christensen, S. L.; Schmid, S. Influence of clamp-widening on the quality factor of nanomechanical silicon nitride resonators. *Journal of Applied Physics* **2019**, *126*, 165108.

(2) Piller, M.; Luhmann, N.; Chien, M.-H.; Schmid, S. Nanoelectromechanical infrared detector. Optical Sensing, Imaging, and Photon Counting: From X-Rays to THz 2019. Vienna, 2019; pp 1–7.

(3) Piller, M.; Sadeghi, P.; West, R. G.; Luhmann, N.; Martini, P.; Hansen, O.; Schmid, S.

Thermal radiation dominated heat transfer in nanomechanical silicon nitride drum resonators. *Applied Physics Letters* **2020**, *117*, 034101.

(4) Szkop, M.; Kliszcz, B.; Kasprzak, A. A. A simple and reproducible protocol of glass surface silanization for TIRF microscopy imaging. *Analytical Biochemistry* **2018**, *549*, 119–123.

(5) Glass, J. A.; Wovchko, E. A.; Yates, J. T. Reaction of methanol with porous silicon. *Surface Science* **1995**, *338*, 125–137.

(6) Kong, M. J.; Lee, S. S.; Lyubovitsky, J.; Bent, S. F. Infrared spectroscopy of methyl groups on silicon. *Chemical Physics Letters* **1996**, *263*, 1–7.

(7) Soethoudt, J.; Crahaij, S.; Conard, T.; Delabie, A. Impact of SiO 2 surface composition on trimethylsilane passivation for area-selective deposition. *Journal of Materials Chemistry C* **2019**, *7*, 11911–11918.

(8) Golub, G. H.; van Loan, C. F. *Matrix Computations*, 4th ed.; The Johns Hopkins University Press: Baltimore, Maryland, USA, 2013; p 755.

(9) Henry, E. R. The use of matrix methods in the modeling of spectroscopic data sets. *Biophysical Journal* **1997**, *72*, 652–673.

(10) Hendler, R. W.; Shrager, R. I. Deconvolutions based on singular value decomposition and the pseudoinverse: a guide for beginners. *Journal of Biochemical and Biophysical Methods* **1994**, *28*, 1–33.

(11) van Wilderen, L. J. G. W.; Lincoln, C. N.; van Thor, J. J. Modelling multi-pulse population dynamics from ultrafast spectroscopy. *PLoS ONE* **2011**, *6*.

(12) Müller, C.; Pascher, T.; Eriksson, A.; Chabera, P.; Uhlig, J. KiMoPack: A python Package for Kinetic Modeling of the Chemical Mechanism. *The Journal of Physical Chemistry A* **2022**, *126*, 4087–4099.

(13) Kollenz, P.; Herten, D. P.; Buckup, T. Unravelling the Kinetic Model of Photochemical Reactions via Deep Learning. *Journal of Physical Chemistry B* **2020**, *124*, 6358–6368.

(14) Motulsky, H.; Christopoulos, A. Fitting models to biological data using linear and nonlinear regression. *Pharmaceutical Statistics* **2005**, *4*.
